# An Academics Guide to Approaching Bioscience Curricula Design: Stakeholders, Material and Assessment Choice, and Employability

**DOI:** 10.1002/bmb.70023

**Published:** 2025-11-13

**Authors:** Kirsten Riches‐Suman, Simon Tweddell

**Affiliations:** ^1^ Scholarship of Teaching and Learning (SoTL) Group, School of Chemistry and Biosciences University of Bradford Bradford UK

**Keywords:** assessment, bioscience education, curriculum design, employability, higher education

## Abstract

The ultimate aim of all higher education programs is to produce work‐ready graduates who can enter a number of career paths. Bioscience graduates are well suited to a multitude of career paths such as research, education or industry. Designing an undergraduate bioscience program that can prepare learners for this multitude of career pathways can be a challenge. Curricula design is a substantive piece of work that is often given to subject specialists who are very familiar with biological science as a subject, but perhaps less well versed in the underpinning pedagogical principles of teaching, learning and assessment. Academics can be left to design curricula alongside their existing teaching, research and administrative duties which leaves little time for thorough research into the theory behind the design process, and how this can be conducted to ensure a focus on employability as well as scientific proficiency. This article aims to provide a “how to” guide for academics who are engaged in designing or redesigning biological science curricula, and is based on experiences of redesigning a Biomedical Science undergraduate degree. It provides an overview of the key considerations to make in the overarching structure of the program, the needs of learners, employers and accrediting bodies, the theory underpinning the comparative strengths and weaknesses of different learning delivery and assessment strategies, and how these can all coalesce to provide a biological curriculum that encourages and enhances diverse postgraduation careers.

## 1 Introduction

There is growing recognition that while bioscience in higher education provides a high‐quality theoretical knowledge of the subject and a critical ability to research, it is not as good at preparing our graduates for the wealth of postgraduation careers that are available to them [1]. Such career paths include bioinformatics, science education, science communication, accredited medical laboratory assistants or technicians, and postgraduate research, among others. This article will discuss a “how‐to” guide for academics developing bioscience curricula to promote an emphasis on employability across a breadth of career types without pigeonholing graduates into one or two specialized paths where places are low and competition is high.

**FIGURE 1:**
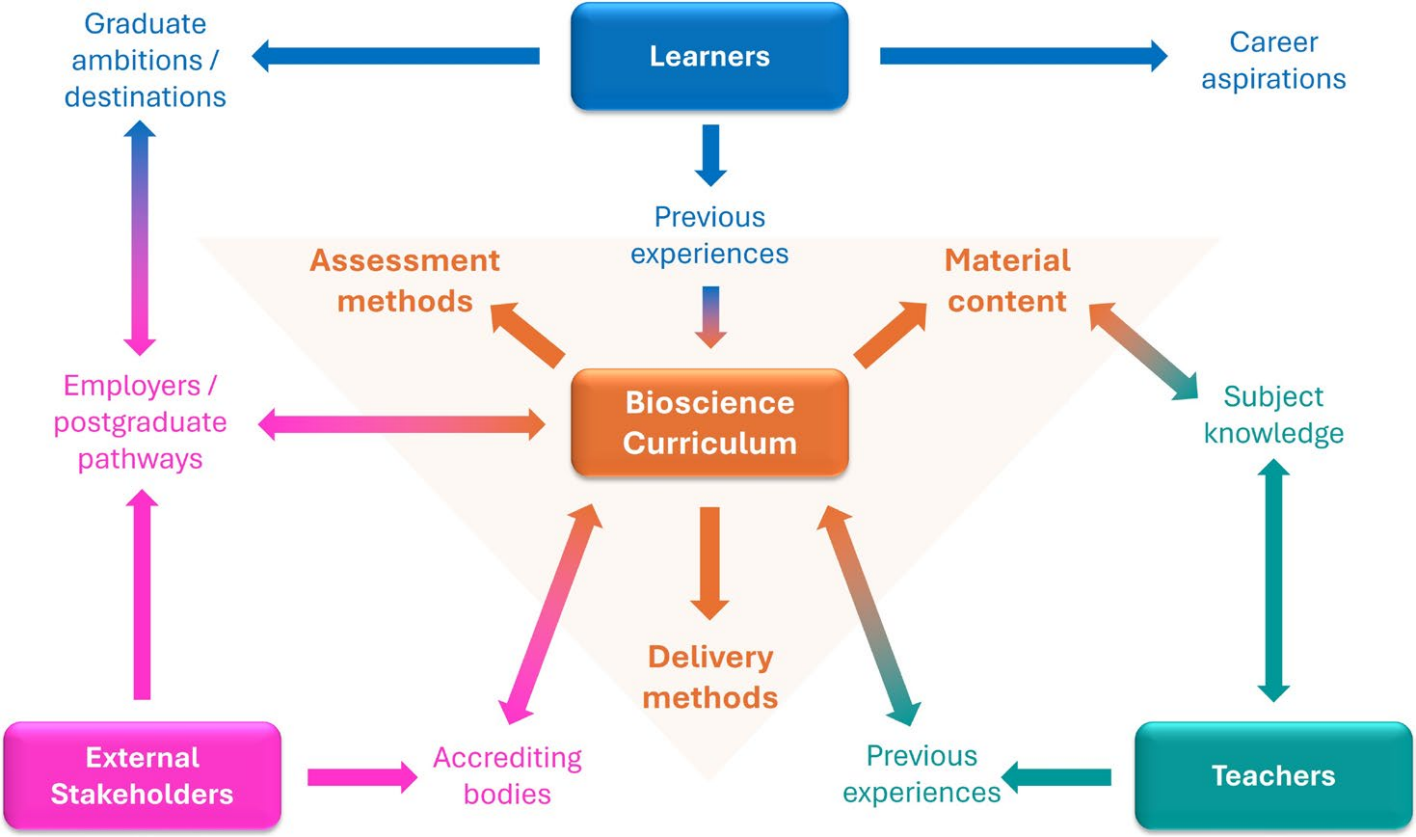
Factors that influence curriculum design. Undergraduate curricula are an amalgamation of the needs of learners, external stakeholders, teachers and the course that you wish to provide. The experiences of learners prior to university influence *how* they learn, while stakeholders such as employers or accrediting bodies influence *what* they learn, and teachers play a central role in delivering the vision of the program. All of this needs to be taken into consideration for the design of teaching materials, delivery methods and means of assessment to ensure that the curriculum will produce well‐rounded, employment‐ready graduates.

Most higher education institutions employ educational developers; individuals who have specific training in designing university programs with the best interests of learners at their core. By their nature, these developers have broad expertise in *learning and teaching* in general and not in the specialisms of each degree course. Thus, the intricacies of specific program design fall to academics who may have very little training in how to go about this substantive task, limited time to commit to it, and limited understanding of the underpinning pedagogical approaches that encourage a cohesive design process [2]. The purpose of this article is to help those academics and provide guidance as to the types of considerations that need to be made to effectively design (or redesign) a bioscience degree to optimize the employability of their graduates. It will encompass the complex and sometimes competing needs of learners, teachers and external stakeholders with material design, delivery and assessment strategies (overviewed in Figure 1). It draws on personal experience of redeveloping the BSc (Hons) Biomedical Science degree at the University of Bradford which received a commendation from our institutional review for its focus on graduate employability. As such, it is reflective of the British education system. However, many of the lessons contained within it are applicable for educational program design across the global landscape.

## 2 Understanding Your Stakeholders

### 2.1 Learners

Your students are at the heart of your curriculum and as such they need to be at the center of your considerations. They are the recipients of your program for which they often pay substantive fees. Therefore, the design of the program needs to be aligned with both what they want—an enjoyable, challenging educational experience—and what they need—a program that will develop their academic knowledge alongside their personal skills so as to become a highly employable graduate. This requires consideration of the learner journey stretching from before they join your institution to where they wish to go afterwards.

#### 2.1.1 What Learners Have Experienced During Further/PostCompulsory Education

Learner experiences in schools and further education colleges are key to understanding where our learners come from, what educational experiences they have had, and how these may feed into their success in higher education. This encompasses:

- The academic and applied knowledge they possess—this is important for understanding the level to pitch your subject content.
- The learning and teaching delivery and assessment strategies they are familiar with—this is important for understanding how they have previously learned and which assessment modalities may be problematic, not for the content they are assessing, but simply because of how content is being assessed.
- The learning environment that they are used to (e.g., small versus large group learning)—this is important for understanding where learners will need guidance on how to learn using a particular method, in addition to learning content.

All of these aspects will affect how learners transition into your degree program.

Further (post‐16) education can vary considerably across the global context, and even within the four nations of the UK. There are questions regarding how further education providers prepare their learners for the rigors and challenges of working life; through the depth and breadth of what they cover, and the level of practical skills that they develop [3]. In the UK, there are four main entry routes into higher education. A‐levels are an example of a more traditionally “academic” educational path whereas Business and Technology Education Council (BTECs), Cambridge Nationals and T‐levels are traditionally “vocational”. There is also a growing international emphasis on “nontraditional” routes into higher education; that is removing the requirement for school/college‐level academic success and offering alternative routes such as Access to Higher Education courses or apprenticeships which are typically taken up by a more diverse learner group, including high proportions of mature, minority ethnic, socioeconomically deprived and/or disabled learners [4]. Indeed, international schemes to broaden this alternative route into education have been proven to be especially beneficial for mature students, who have had a break from education, and socioeconomically deprived students [5].

A‐levels are predominantly classroom‐based with a combination of didactic teaching, hands‐on practical work, problem‐solving/applied understanding group work, and formative multiple‐choice questions (MCQs). A‐levels tend to be assessed almost completely at end‐of‐stage via closed book, time‐bound examinations which comprise a combination of MCQs, data analysis and interpretation, and knowledge and understanding short‐answer questions. One of the benefits of this is that websites are freely available with past exam papers and assessment strategies which help academics (who have often been out of further education for many years) understand what experience their learners have had. Questions require more depth of knowledge than equivalent vocational qualifications but the programs do not offer as much hands‐on practical content.

Vocational qualifications include BTEC, Cambridge Technicals and T‐levels. These courses tend to have more hands‐on laboratory experience and are focused more on the application of knowledge rather than retention. In 2021, T‐Levels in science subjects were launched [6]. These will straddle BTECs and A‐Levels and will eventually replace the old BTEC qualifications. While students entering via this route should have more practical skills, the reduced emphasis on core biological and chemical concepts could prove problematic.

Access to Higher Education courses have a broad syllabus that covers the basic principles of chemistry and cell biology, and they also provide training on transferrable skills such as communication and critical thinking. Their assessment strategy includes essays, presentations, reports, laboratory practicals, research investigations and two end‐of‐year examinations, which is similar to experiences students will encounter during undergraduate degree programs.

#### 2.1.2 Why Learners Apply to Your Institution

Many factors influence why learners choose particular HE providers. Location, reputation, programmatic accreditation, facilities, and the views of parents and friends are all important factors in choosing a program, as well as the course curriculum itself [7, 8].

If you are redeveloping an existing degree program, it is useful to consult with existing students on what their motivations for attending your institution are. For example, is it because it is local? A “local” university can be very beneficial for adult learners who may not have the flexibility to move due to caring responsibilities [9]. Given the increasing cost of living and socioeconomic factors, access to local universities where students can live with family and/or commute by foot could also increase access to education [10] and could keep universities afloat during challenging financial times. Programmatic accreditation remains a significant draw for learners as it immediately opens up access to postgraduate career routes, and will be discussed further in Section 2.3.

#### 2.1.3 Employability Aspirations and Destinations

Over half of university applicants consider graduate employment rates to be a good indicator of teaching quality [11]. These statistics are readily available on degree program comparison websites. Thus, considering the career aspirations that students start their degree programs with, and how this compares with graduate outcomes, is an important consideration. If you are redeveloping an existing program, consider looking at higher education leavers data to determine what career paths most of your graduates progress on to. This will help you to align your learning material and methods to careers that are proven to be attractive to your graduates.

#### 2.1.4 Collecting Information From Learners

If you are redeveloping an existing program, your current learners are a rich source of guidance as to what works, what doesn't, and what can be improved. Thus, having meaningful, two‐way open discussions with current learners should be embraced. For example, 1–2–1 or small group discussions with current learners across the different years of the program, followed by a thematic analysis of the emerging areas for concern or celebration, alongside consultation with student‐staff liaison committees, can be informative. Where learners are comfortable and open, this can also give direct insight into how well they feel their degree is preparing them for employment and what they may perceive as lacking. However, care must be taken to ensure the learners feel that they are being listened to, and that their perspectives are valid and valued [12]. For learners who do not wish to engage with direct feedback, anonymous end‐of‐semester surveys can also be used. Most institutions have these as a ready source of feedback for the educational team and they can also be mined for indicators on things that work well, and things that don't, across the cohort. A strength is that they are anonymous and so responses are more likely to be honest and detailed [13]. Limitations include the fact that free‐form answer questions can degenerate into personal insults against academics rather than focusing on the content and methods of delivery and assessment, so care should be taken in framing the questions and optionality for response [14].

If you are designing a completely new program of study, finding this information can be more challenging. In this instance, expert guidance in market research should be provided (or sought) from the institution itself as this is beyond the capacity of a subject‐specialist academic to perform in isolation.

### 2.2 Employers

The ultimate aim of any degree program is to equip its graduates with the skills and knowledge needed to gain meaningful employment. Graduate attributes are key for ensuring any degree program provides learners with employability skills. Importantly, we as academics rarely have all the insight needed for graduate attributes and so engaging with employers is necessary to fully appreciate the transferrable skills that need to be embedded within the program to enhance employability. An external advisory board to inform curricula design, composed of individuals from local industry employers can be highly desirable [15]. Employer involvement with curricula design is positively linked to employability outcomes [16], and more specifically in the definitions of qualification standards and curriculum fluidity to meet emerging employer‐related demands [17]. Embedding employability skills within modules is also more beneficial than passively offering careers advice external to the core program, which has implications for the design of learning and assessment resources and will be discussed in further sections [18].

In the absence of employer advisory boards, the needs and wants of future employers can be assessed by auditing the jobs market [19]. For example, interrogating employment advertising websites where your program (e.g., biomedical or biological degree) is listed as a requirement can yield a wealth of information. These tend to show a need for transferable skills rather than academic knowledge, but of course this varies from position to position. There are also governmental and private employment analysis databases that can provide an overview of the graduate attributes and skills that are desirable for different given professions such as laboratory technician, research assistant, science educator etc. Examples include O*NET OnLine (US Department of Labor), The Burning Glass Institute (US) and LinkedIn's Future of Skills (global). Some of these can also predict in which areas the graduate employment market will grow, allowing you to design curricula and programs that prioritize skills development with the future employment landscape in mind.

Given that many life sciences students go on to work in research in one form or another, including PhD study [20], an audit of PhD supervisors can also be conducted. Again, specific areas of knowledge (e.g., vascular biology, microbiology) are rarely mentioned as a graduate attribute as this would be taught during the program of study. From informal discussions with PhD supervisors, transferable skills were much more important including critical thinking, scientific curiosity, initiative, writing and English language skills. Thus, it is important to embed these within the curriculum.

### 2.3 Accrediting Bodies

Programmatic accreditation by external professional bodies can be a strong draw to students. Not only does it provide evidence of external scrutiny, it also provides a marker of approval of program standards, content and quality assurance [21, 22] and can be a gatekeeper into particular employment routes. For example, in the UK graduates hoping to work in a National Health Service (NHS) laboratory as a biomedical scientist have to have completed a degree with programmatic accreditation from the Institute of Biomedical Science (IBMS) and approved by the Health and Care Professions Council (www.healthcareers.nhs.uk). If the degree program does not have these external accreditations, then individuals would have to complete accredited study after the completion of their degree before they could apply for the preferred position. This can have cost implications as well as delaying their entry into their chosen career paths.

Other programmatic accrediting bodies focus more on transferable skills; for example the Royal Society of Biology (RSB). The RSB accredits degrees that fit within the general remit of “Biology”. Due to their broad portfolio, they do not have a series of benchmark statements for specific academic programs and instead are more focused on transferable skills and employability. Furthermore, RSB accreditation is endorsed by large life science industrial providers in the UK and so receiving accreditation can provide an additional draw for incoming applicants by validating the employability focus of the provision.

### 2.4 Curricular Consultants

While the minutiae of curriculum design are often left to the subject‐specialist academics, curricular consultants can help in the development of the curriculum. This could be an internal educational developer from within your institution who joins the curriculum development team. It could also involve external consultants such as employers, external experts in your pedagogy, other stakeholder representatives, e.g., NHS, patient or other healthcare groups, alumni or professional body representatives who join a Curriculum Steering Group to inform, advise and steer the work of the internal curriculum development team.

## 3 Curriculum Design

### 3.1 Overarching Principles for Curriculum Design

Curricula design should be informed by educational evidence and educational research. Educational practice isn't always aligned with the evidence [23]. There are many different approaches to curriculum frameworks, including linear, spiral and network approaches [2, 24]. Each has its own strengths and weaknesses and the applicability of each is individual to the course that you are designing, your learners, and your academics. As such, this report points the reader to useful articles on the different types of curriculum frameworks above, but will focus on the spiral approach as this was the one that we chose for our program. Spiral curricula, where concepts are revisited more than once to consolidate knowledge and understanding, increasing in depth, breadth and complexity on each visit, have been proven to enhance student learning and to help create links between different modules both within and across stages [25] and have a long evidence base for success in scientific subjects [24].

In our institution, we teach across three academic years (also termed stages), with 6 modules within each academic stage. We performed a thematic analysis of the accrediting body subject requirements to determine 6 themes that could be revisited, with increasing levels of complexity, at each stage. In general, Stage 1 modules laid foundational concepts and ensured that all learners, independently of the route from which they joined us, had the same baseline knowledge and understanding which we could then build upon in Stage 2 with more application of knowledge (rather than simply retention). Stage 3 then built on these foundations with subject‐specialist knowledge and much more emphasis on application of understanding, problem‐solving and employment‐centered skills.

For each module, we considered four key aspects to ensure constructive alignment [26]. We also utilized a “backward design” approach [27], whereby we first considered the learning outcomes and ensured the other three aspects were designed with the learning outcomes in mind:

- Learning outcomes: What are the learning outcomes of the module and how do these embed your module aims and assessments strategies?
- Aims, outline syllabus and learning resources: What subject material needs to be covered within this module? What will a learner who has completed this module achieve? Why should they want to study this module?
- Delivery: What is the most effective way to deliver this content? How will learners be engaged? How do we ensure learners take deep approaches to learning? What should our learning and teaching strategy be to ensure learners meet the outcomes relating to both academic knowledge and understanding and transferable skills?
- Assessment structure: What assessment modalities have been chosen, and how are these related to skills that graduates will require in their future careers? How have you prepared your learners for these assessments? What are the opportunities for formative assessment? How do we optimize feedback and feedforward? Are the assessments inclusive?

The subject content will depend on your individual program and as such we will not specify here what should or should not be included in terms of subject material. Instead, we overview the different methods of delivery and assessment and their benefits or detriments, before suggesting how employability can be embedded within the course material, learning and teaching and assessment strategies for your offering.

#### 3.1.1 Curriculum Delivery Methods

Didactic learning is prevalent in STEM topics to “bank” large volumes of knowledge [28, 29] and can be useful with large cohorts from diverse backgrounds to ensure all learners are exposed to the same foundational concepts. This method was challenged by widespread university lockdowns and a move to online learning during the pandemic. Following the reopening of campuses, didactic face‐to‐face lectures have also returned, although attempts to maintain a blended learning delivery (a combination of face‐to‐face and online provision) have been made. Previous studies have highlighted that blended approaches, with a combination of hands‐on real time and asynchronous activities can enhance learning and self‐directed skills [30]. Interestingly, there was no difference in achievement between students taught material exclusively online versus traditional face‐to‐face lectures, although isolation and a lack of engagement have been noted with the former [29].

In contrast to didactic methods, studies have shown that the quality of student learning is better when active learning techniques are used, and indeed an over‐reliance on didactic lectures in STEM subjects can reduce pass marks [31]. Active learning moves the learning experience away from memorization and instead encourages application and inquiry which are more desirable for future careers [32]. One problem that can be encountered with introducing an active learning focus is problems with “buy‐in” from educators who are wedded to didactic styles because this is all they have experience of (both as a learner and as an educator; [33]). Furthermore, many educators are unclear on what active learning actually is. Active learning covers a vast array of techniques including, but not limited to, student question and response systems (quizzes, polls, etc.), problem solving and case studies, flipped classroom approaches where learners direct the session rather than the lecturer, think‐pair‐share activities, pre‐ and/or postlecture quizzes, reading or videos [32].

Many of these are easy to incorporate with minimal effort into existing didactic lectures to improve student engagement. In‐class quizzes are well received by learners and have a pedagogical underpinning [34, 35]. Inclusion of one or more modules of Team‐Based Learning can also promote group work and enhance student outcomes [36]. While this approach has clearly been evidenced to improve deep‐learning skills [37] it can be a difficult transition for students to make as it is unlike anything they have experienced prior to university. Thus, care should be taken in how to ease the transition into any of these learning methods in a low‐stakes, supportive environment, preferably early on in the university journey.

STEM topics such as bioscience are by necessity, practical programs. Practical and applied skills are particularly important for external programmatic accreditation, employers, and are increasingly being used for clinical and analytical settings [38]. Practicals throughout the first 2 years of a degree program are also, theoretically, preparing learners for the immersive research project that life sciences degrees typically culminate in. Undergraduate practicals are traditionally taught through “recipe‐based” or “cookbook” styles which don't necessarily encourage the related (and desirable) skills of hypothesis formation, experimental design and unexpected experimental outcomes [39, 40]. However, it is common in large cohorts to teach in this way.

Some institutions create laboratory‐*focussed* modules; however these are not exclusively laboratory‐*based*. From examples in the literature, there is a preference for a four‐week laboratory practical process based around a defined research question (e.g., gene expression, endocytosis, enzyme activity; [39, 40, 41]). This is underpinned by prelearning on theory and practical skills and is followed by data interpretation and critical thinking.

Embedding all practicals within one single module and cross‐linking them with academic content in all other modules, may help students to situate practical learning in one place. However, the challenges with integrating very disparate practicals—e.g., from the basics of pipetting to streak plating to histochemistry and qPCR—into a single module would be extremely challenging without resorting to cookbook teaching. Instead, embedding practical elements in all taught modules can provide a cohesive learning experience.

#### 3.1.2 Delivery Timing

One aspect of delivery that requires careful attention is the timing of teaching sessions. Often, teaching sessions take place within the normal working day, e.g., 9 am till 5 pm, Monday and Friday. This may well be ideal for full‐time learners, straight out of further education, who may not have any other responsibilities. However, there is a growing desire to increase the number of mature learners who commonly have many different things competing for their time. For example, they may work part‐time, or have young children that mean their studies need to fit around school drop‐off and pick‐up times. Widening participation for these diverse learners is receiving increased attention.

The COVID‐19 pandemic necessitated a switch to online learning. Most institutions—and indeed, most academics—are now familiar with how to deliver lectures remotely which facilitates asynchronous delivery that can be more accessible to diverse learners. Hybrid methods, where learners access the majority of their learning material in their own time but attend campus for short, fixed periods of time for hands‐on laboratory training, have been adopted by some HE providers even when they are no longer restricted by lockdowns. However, care must be taken to ensure that those from socioeconomically deprived backgrounds are not disadvantaged due to difficulties in accessing adequate technology [42]. Furthermore, the uptake of online learning in countries where online access is less reliable, may limit asynchronous or hybrid delivery in the global context [43]. Asynchronous delivery, where material is accessed at a learner's preferred time and pace, can be beneficial for both traditional and mature students alike due to its flexibility and accommodation of “life” outside of university. However, it can also promote isolation and reduce the social aspect of learning, which would need to be actively managed [44].

One interesting aspect of hybrid delivery is how it can be adapted for apprenticeships. In the UK, apprenticeships are a form of learning that couples on‐the‐job training with studying for a university qualification. In our institution, we offer an Applied Biomedical Science integrated degree (in parallel to our standard Biomedical Science degree), which is an apprenticeship designed for existing NHS pathology laboratory workers. Using asynchronous delivery and only requiring campus attendance for specific hands‐on laboratory sessions has made the learning experience more accessible for NHS workers. It has also eased constraints on timetabling for co‐taught sessions—in the past, when asynchronous delivery was not standard, apprentices attended campus on day release which meant loading all co‐taught sessions for both degrees into a single, intensive day that was a regular source of complaints for nonapprentice learners.

Offering individual modules that are taught asynchronously, or synchronously but in nontraditional hours (e.g., evenings, weekends) can also enable nontraditional learners to up‐skill at times convenient to them. For example, biomedical science learners who have undertaken a non‐IBMS‐accredited degree have to take additional accredited modules in order to work in the NHS (http://ibms.org). Designing a curriculum that can be adapted to asynchronous delivery or evening classes would be beneficial for employment prospects in these cases.

#### 3.1.3 Assessment Structure and Methodology

Learners (and sometimes educators) approach assessment as “learning‐to‐pass” rather than “learning‐to‐practice” [45], which can lead both educators and learners to view assessment as a tick‐box exercise [46]. Because of this, several frameworks have been developed to guide assessment strategies that reward application and engagement rather than simply regurgitation. Similar to the section on curriculum design, this article will not go into depth on different assessment frameworks. Rather, we point the reader to useful toolkits on a variety of frameworks such as that from AdvanceHE [46] or program assessment strategies [47] so that the reader can see what is most appropriate for their course and institution. Instead, what we will focus on here is the pedagogical reasoning behind different assessment strategies.

One key aspect of assessment that should be embraced is formative assessments. These are an essential part of modern curricula and are an effective way of giving valued feedback and feedforward [48]. It is also a matter of fairness—assessments need to evaluate knowledge, understanding and critique and should not be influenced by students panicking that they are undertaking a method of assessment they are not familiar with. Thus, all assessment modalities must have a formative version prior to their first use. While student engagement in formative assessment can be limited if they believe it “doesn't count” towards their final mark [49], we need to enforce the mindset that formative assessments are compulsory and are of great benefit to the learner. Similarly, they demonstrate active student engagement with well‐written rubrics [50] and are a means to integrating assessment literacy into course design.

Assessments can be defined as assessment *for* learning, assessments *as* learning and assessments *of* learning [51]. The former two are developmental and most likely to be formative taking place during the semester, while the latter is summative for which marks or grades are attributed and are more likely to occur at the end of the semester.

- Assessment for learning help teachers and students identify learning gaps during teaching, guiding improvements and adjustments.
- Assessment as learning empowers students to reflect on their own progress, fostering self‐awareness and independent learning skills.
- Assessment of learning is used to evaluate students' achievements at the end of a learning period, often through tests, examinations or final projects and contributes towards the final grades of the course or program.

Every academic program should develop an assessment strategy which should constructively align with the learning outcomes of the program, i.e., to ensure you are assessing the learning outcomes and that the assessments chosen are best placed to assess them effectively. The assessment strategy should also align with the learning and teaching strategy to ensure that students are being prepared effectively for the assessments. Learning outcomes should be informed by societal needs, professional and statutory body requirements and informed by employer requirements. As suggested in Section 2.2, an external steering group that has employer, student, alumni and professional body representation could be formed to help steer the development of the curriculum.

Examples of summative assessments are as follows.

Traditional end of term, time‐bound, closed‐book assessments are similar to the format that learners experience prior to attending University. They promote deep learning [52], however recent studies have demonstrated that they can have a disproportionally negative impact on learners with disabilities or exam anxiety [53]. Given the increasing recognition of learner‐specific profiles, time‐bound closed‐book assessment may not be the most inclusive format. However, these types of assessment do offer many benefits. Students do not take open‐book assessments as seriously as closed‐book ones and thus do not prepare adequately, which is reflected in lower grades [52, 54]. Essay‐based questions can give learners the opportunity to demonstrate the complexity of their knowledge and understanding [55].

MCQ‐based assessment is traditionally used for memory recall but can be adapted to assess cognitive processing and understanding [56] and has been shown to be just as effective at assessing understanding as essays are [57]. Contrary to popular contemporary belief, they are not a modality that encourages exclusively surface learning. Item analysis (often available through virtual learning environments) provides good feedback to educators on how effective their MCQ design has been [58], and MCQs can effectively identify which learners are struggling early on in their higher education journey which enables us to put processes in place for individual learner support [59]. Furthermore, it is an assessment method that students are familiar with irrespective of their route into higher education and thus will not add complexities from a lack of assessment literacy.

Laboratory reports are possibly the most directly relevant assessment strategy for learners who will go on to work in research or industry, as they mirror in some ways the documentation that they will have to produce during their working life. They require an understanding of the underpinning theory of the practical, an understanding and proficiency of the technical requirements, an ability to collect data and comply with regulations on quality control, and an understanding of the surrounding context within which their results sit. Furthermore, embedding laboratory reports throughout a program can prepare students for producing the capstone dissertation which often comprises the most significant single assessment within scientific programs. Dissertations are often considered as the “hallmark” of the final year degree program and can be very useful in transforming learners' understandings of concepts from the theoretical into being applicable to their own lives [60].

Using case‐based learning and assessment promotes deeper understanding and better prepares learners for working life postgraduation [61]. Similarly, problem‐based learning and assessment are well recognized to address deeper learning and applicability skills as well as critical thinking to solve real‐world problems [62]. Both are readily adaptable to bioscience topics. Thus, as well as being a dynamic and engaging assessment modality, they prepare learners for the world of work when they can apply their problem‐solving and critical thinking skills to work‐based issues.

Visual or verbal‐based assessments appeal to different learner types and assess in a very different way from written assignments. Engaging in presentations such as journal clubs improves the critical interpretation and understanding of basic research and also develops confidence and communication skills [63, 64]. An additional benefit of presentations (either alone or as part of a group) is that they lend themselves to peer assessment which can be very beneficial for learning. It can improve attainment over and above assessment from educators alone with the benefit being stronger if peer assessment is regularly used [65]. Moreover, it forces learners to engage with rubrics which is something that despite being repeatedly advised to do so, learners often do not [50]. However, it has also been found that assessing “employability” skills such as presentation skills has a negligible impact on graduate outcomes [16].

Individual learner‐educator vivas are a recognized modality for assessing deeper understanding and communication skills and students tend to prepare well for them [66]. While learners can find them nerve‐wracking, the 1–2–1 nature of vivas lends itself well to educators being able to facilitate a relaxed assessment environment. Debates are core to many degrees in social and political sciences but are comparatively less used in biosciences. There is growing recognition of the importance of debates in considering how research impacts on society and thus debating is to be encouraged [67]. Studies have shown that using debates in higher education enhances critical thinking, deep learning and contextualization of material for both learners and educators [68] and important scientific concepts such as ethics are ideally suited.

A continual cycle of assessment, often referred to as closing the loop, is a foundational principle in curriculum design that ensures educational programs remain responsive, effective, and aligned with intended learning outcomes. This cyclical process involves the systematic collection of data on student feedback and performance, through both formative and summative assessments, and staff reflections and feedback. This is followed by critical analysis and the implementation of targeted improvements to learning, teaching and assessment strategies, course content, and learning environments. The effectiveness of these changes is then reassessed, creating a feedback loop that promotes continuous enhancement of the curriculum [69]. For each individual degree program, it is likely that a unique combination of different delivery and assessment methodologies will be most appropriate. The information discussed above will help subject‐specialist academics to understand the pedagogical strengths and weaknesses of each methodology.

#### 3.1.4 Embedding Employability in Syllabus and Material Design

Bioscience graduates have a large array of potential careers open to them [20]. For UK Biomedical Science specifically, there is the route into the NHS. Other common career paths include postgraduate research, industrial or contract research, laboratory technical positions, science teaching and science communication to name but a few. Graduates should be equipped with the knowledge and skills that allow them to thrive not only in their current jobs, but also with the adaptability to be able to fulfill future career needs.

In many instances, learners struggle to see how their course has equipped them with the transferrable skills that are so desirable to employers and are commonly listed as “essential criteria” in job recruitment. A recent study identified that using questionnaires on how students have developed their critical thinking, teamwork and communication skills in their final year helps them to appreciate these facets, understand their employability and enhance their desire to continue their careers in the scientific industry [70]. Furthermore, creativity, enterprise and entrepreneurship are core skills for graduate employability, and in some cases are more desirable than communication skills [15]. The concept of creativity is very difficult for students to grasp [71], even though scientific enquiry is by its very nature a creative process. A guided reflective process on this can therefore be of benefit to learners [72].

Undoubtedly, one of the most desirable qualities of graduates in employment (and indeed life in general), is the ability to problem solve. This life‐long learning skill includes the ability to innovate, to come up with creative solutions to work‐based problems, and to be able to problem‐solve both independently and as part of a team. Creative problem‐solving training has been shown to improve problem‐solving skills, achievement and extroversion in research and development workplace settings [73], and embedding these within the preworkplace university setting can be advantageous. However, the difficulty with embedding this in curricular design is that problem‐solving is an inherently interpersonal and complex process. Models such as the four‐component instructional design model [74] can be easily adapted into spiral curricula, and can be effective for both practical‐based problem‐solving (e.g., experimental optimization) and for theoretical‐based problem‐solving (e.g., analysis and interpretation of clinical test results).

This can also help with capstone projects. Most learners have the opportunity to conduct original research within their final year projects. However, this offers only a small taster into conducting and analyzing research and can be more supervisor‐directed than learner‐directed, which does not provide optimal preparation for postgraduate study and the subsequent career paths that this can lead to [1]. Embedding learner‐directed research early on in their university course can promote these skills more and improve the independent thinking and critical interpretation skills that are valuable to a wide variety of career paths [75]. Furthermore, embedding placements (either through short placements within teaching time, or intercalating years) within the degree program can be invaluable for enhancing employability postgraduation [16, 17]. The traditional “academic” career pathway—undergraduate to doctoral study to academic and research lead—in science is oversaturated. Competition for postgraduate study is high, mental wellbeing is low, and the prospect of becoming a full‐time academic is very low [76]. It is important therefore to prepare our graduates for alternative career paths that are also very competitive.

The term “industry” in this context seems to be a catch‐all term for any nonacademic scientific career and thus is inherently broad. Employers have reported a need for universities to embed industry‐specific content into curricula; for example commercialization and the importance of regulations [77]. Furthermore, industries have their fingers on the pulse of what the future job market demands will be; for example if there are developments that will require greater critical thinking skills, and so engaging with them throughout the process can be key to future‐proofing not only your course, but your graduates as well [78]. These are additional reasons why embracing employer perspectives at the very start of curricula design is key in best serving your learners for their future careers. Including some pedagogical material in how to teach science can benefit those students who wish to go on to postgraduate study in secondary education and there are models to help incorporate this into a scientific program [79].

An interesting aspect of assessment that can promote employability is the inclusion of peer assessment. While the benefit of peer assessment in attainment may be small, it helps to prepare learners for working life where peer evaluation of performance is commonplace [80]. Self‐ and peer assessment immerses learners in the purpose of assessment, the means of assessment, and allows them to hone their evaluative skills and judgment of professional standards [81, 82], all of which are beneficial for employability. It can also promote a sense of community [83] through shared experiences.

One final thought on employer engagement, is that in some instances a “sense of entitlement” has been reported, with learners not appreciating that although a degree can open the door to their employment, it is not enough to keep them in their job—they have to perform to the appropriate level as well [84]. Indeed, a very recent study highlighted increased academic entitlement in postpandemic learners [85] which is often linked to poorer academic practices and outcomes [86]. This emphasizes the need for universities to reinforce the fact that performance is benchmarked to national standards and the effort has to be put in to get the grades; much like performance in employment is related to employer standards, and simply showing up with a certificate is not enough. Graduates who can demonstrate creativity, adaptability and an ability to engage with life‐long learning are therefore very desirable.

## 4 Conclusion

The purpose of this article was to help guide busy, subject‐specialist academics with a “how to” guide on how to approach curriculum design with employability in mind, and to provide a pedagogical underpinning of different delivery and assessment modalities (overviewed in Figure 1). When time is short, it can be tempting to go down the “we will teach it this way because that's the way it has always been done” path, rather than engaging with literature to see if that is indeed the right path. Designing a new program (or redesigning an existing one) is a substantive job as learners who ultimately take the program of study will be paying thousands of pounds throughout their study. This article has discussed the considerations that need to be made to provide a considered curriculum that will produce well‐rounded, workplace‐ready graduates who have the knowledge base and the transferable skills needed to be successful in whatever career path they choose.

## Author Contributions

K.R.‐S. conceived the manuscript, drafted and edited all sections. S.T. contributed significantly to the section on Curriculum Design and provided constructive feedback on the entirety of the manuscript. Both authors read and approved the final manuscript.

## Conflicts of Interest

The authors declare no conflicts of interest.

## Data Availability

Data sharing is not applicable to this article as no datasets were generated or analyzed during the current study.
